# Fighting microbial drug resistance: a primer on the role of evolutionary biology in public health

**DOI:** 10.1111/eva.12254

**Published:** 2015-03-23

**Authors:** Gabriel G Perron, R Fredrik Inglis, Pleuni S Pennings, Sarah Cobey

**Affiliations:** 1 Department of Biology, Bard CollegeAnnandale-on-Hudson, NY, USA; 2 Department of Biology, Washington University in St. LouisSt. Louis, MO, USA; 3 Department of Biology, San Francisco State UniversitySan Francisco, CA, USA; 4 Department of Ecology and Evolution, University of ChicagoChicago, IL, USA

**Keywords:** adaptation, antibiotic resistance, ecology, epidemiology, experimental evolution, genomics, HIV, malaria, population genetics

## Abstract

Although microbes have been evolving resistance to antimicrobials for millennia, the spread of resistance in pathogen populations calls for the development of new drugs and treatment strategies. We propose that successful, long-term resistance management requires a better understanding of how resistance evolves in the first place. This is an opportunity for evolutionary biologists to engage in public health, a collaboration that has substantial precedent. Resistance evolution has been an important tool for developing and testing evolutionary theory, especially theory related to the genetic basis of new traits and constraints on adaptation. The present era is no exception. The articles in this issue highlight the breadth of current research on resistance evolution and also its challenges. In this introduction, we review the conceptual advances that have been achieved from studying resistance evolution and describe a path forward.

## Introduction

The evolution of microbial pathogens able to resist antimicrobials treatments is one of the most pressing public health crises (Maynard Smith et al. 2000; Palumbi 2001; Bush et al. 2011; CDC 2013; Davies et al. 2013; Nature 2013). Indeed, the European Centre for Disease Prevention and Control (ECDC) estimates that each year, 25 000 people in Europe die directly from drug-resistant bacterial infections (ECDC 2011), while recent estimates provided by the British government suggest that more than half a million people die worldwide from resistant infections (Davies et al. 2013). Antibiotic resistance also imposes a significant financial burden on world economies, with the USA alone spending an estimated $35 billion per annum on resistant infections (CDC 2013). Although what has now been described as the ‘antibiotic crisis’ may seem a relatively recent phenomenon (Cohen 1992; Neu 1992; Bush et al. 2011), Alexander Fleming predicted the evolution of antibiotic-resistant bacteria soon after his discovery of penicillin in 1929.

Drug resistance appears to be a simple case of adaptation via natural selection: The use of antimicrobials in medicine and agriculture imposes selective pressure on microorganisms, favoring the persistence and spread of bacteria with resistant phenotypes (MacLean et al. 2010a). Perhaps unsurprisingly, the quest for a ‘magic bullet’ continues to disappoint (Ehrlich 1912; Cole 2014; Ling et al. 2015). Antibiotics that were once thought to be evolution proof have failed to live up to their promises (Perron et al. 2006; Sun et al. 2009b). Worse, commercial investment into antibiotic discovery is running dry (Livermore 2011). Not only is antibiotic research and development expensive, but the rapid evolution of resistance also reduces expected market returns (Falagas et al. 2006; Payne et al. 2007). As governments, public health organizations, and healthcare workers fight the spread of drug-resistant bacterial infections, it is worth considering how evolutionary biologists, who have long used the evolution of antibiotic resistance as a model system, can help improving strategies to manage antibiotic resistance.

In this special issue of *Evolutionary Applications*, we discuss recent work on antimicrobial resistance evolution, highlighting how this research is of immediate relevance to public health and of interest to ecology and evolution. The contributions in this issue reflect the increasing breadth and depth of expertise in this field. In this article, we introduce the theoretical underpinnings of these contributions and describe how the study of drug resistance has advanced our understanding of evolutionary biology over the past sixty years. We also speculate on directions the field may take in the future.

## Drug resistance: an ecological problem

From a human perspective, microbial drug resistance is the process by which pathogens evolve defenses against the pharmacological compounds used to treat infections. However, antibiotics belong to a wider category of molecules known as secondary metabolites – a group of nonessential molecules produced from primary metabolites (Bu'Lock 1961). In fact, most antibiotics are multifunctional and include pigments, toxins, and effectors of various kinds (Demain 1998). Clinical antibiotics are frequently isolated from saprophytic microbes such as filamentous actinomycetes and fungi, which compete intensely in terrestrial habitats (Gottlieb 1976). In their natural state, a majority of microorganisms produce antibiotics in nonlethal trace amounts (Gottlieb 1976; Kümmerer 2001; Kummerer 2009). For this reason, the primary role of antibiotics in natural environments is a matter of active debate (Yim et al. 2007; Martinez 2008; Davies and Davies 2010). Most antibiotics target key cellular processes, such as DNA replication or protein translation, which might affect bacterial growth even at low concentrations – for a comprehensive review, see Walsh (2003). Therefore, even small concentrations of antibiotics could confer a local fitness advantage for the producer and impose selective pressure for resistance in susceptible competitors.

### Antibiotic resistance in the wild

Antibiotic resistance has been isolated in virtually all environments (D'Costa et al. 2006; Perron et al. 2008c, 2015; Allen et al. 2009; Sommer et al. 2009; Nesme et al. 2014). The total diversity of resistance genes in the wild is hard to estimate (Nesme et al. 2014). The Comprehensive Antibiotic Resistance Database lists over 3000 annotated resistance genes, a number that is constantly increasing (McArthur et al. 2013). Moreover, every functional metagenomics screen of microbial populations, which can identify resistance genes in unculturable bacteria (Allen et al. 2010; Pehrsson et al. 2013), unveiled a plethora of novel resistance genes (Sommer et al. 2009; Forsberg et al. 2012; Perron et al. 2015).

Bacteria have evolved diverse strategies to protect themselves from the effects of antibiotics. Some of the most common resistance mechanisms include efflux pumps, which reduce the concentration of antibiotics inside the cell, and enzymes that degrade or modify antibiotics – for a comprehensive review, see Walsh (2003). In nature, antibiotic resistance might evolve under several scenarios. At the very least, microorganisms producing an antibiotic need to protect themselves against its toxic effects, and they may develop complex mechanisms to protect their cellular target(s) (Davies and Benveniste 1974; Davies and Davies 2010). Antibiotic resistance could also evolve from environmental selective pressures, such as the presence of heavy metals (Piddock 2006; Gullberg et al. 2014) or inter- or intradomain signaling (Yim et al. 2007; Aminov 2009). Finally, although native antibiotics in soils rarely reach high concentrations (Gottlieb 1976; Yim et al. 2007; Davies and Davies 2010), ‘subclinical’ concentrations may exert sufficient selective pressures to drive evolution (Gullberg et al. 2011, 2014). Genomic and functional analyses of ancient permafrost soils revealed the presence of antibiotic resistance in bacteria dating back thousands of years (D'Costa et al. 2011; Perron et al. 2015). Phylogenetic analyses of β-lactamase genes suggest that resistance genes have coevolved with antibiotic genes in bacteria for millions of years (Hall and Barlow 2004; Aminov and Mackie 2007).

Although resistance is both ancient and natural, modern practices in medicine and agriculture greatly impacted the distribution of resistance genes in the human environment – see Baquero et al. (2015) and Chang et al. (2015) in this issue for an in-depth discussion of the topic. Anthropogenic use of antibiotics has increased the number of resistance genes in environmental reservoirs, which in turn has increased the spread of antibiotic resistance in human populations (Forsberg et al. 2012; Wellington et al. 2013; Nesme et al. 2014). Using case studies, Chang et al. (2015) identify three main routes for the transmission of antibiotic resistance from agriculture to the clinic: direct infections, evolution of the ability of resistant pathogens to infect human hosts, and the transfer of resistance genes to human pathogens. Although quantifying the relative roles of these transmission routes remains difficult (particularly in the case of resistance gene transmission), the scale of agricultural antibiotic usage is worrying in light of recent episodes of transmitted resistance (Wang et al. 2012; Spoor et al. 2013). The authors conclude that managing resistance transmission from agricultural to clinical settings requires better surveillance of antibiotic use in animals to identify potential sources of resistance.

Antibiotic pollution from the release of antibiotic waste and manufacturing products into the water supply has also increased the prevalence of antibiotic resistance genes in urban and natural environments (Gaze et al. 2011; Martinez 2012; Amos et al. 2014). A microbiological survey of soil cores around Europe showed a marked rise in the frequency of resistance genes over the past century (Knapp et al. 2010). Antibiotic-resistant bacteria from urban areas can spread far through human travel, wind and precipitation (Christner et al. 2008), and migratory birds (Sjolund et al. 2008). The relative contributions of environmental reservoirs to resistance in pathogenic bacteria remain an active field of research (Forsberg et al. 2012; Finley et al. 2013; Wellington et al. 2013; Graham et al. 2014).

## Drug resistance: an evolutionary problem

From a medical perspective, antimicrobial resistance is a relatively recent evolutionary problem. However, given the ubiquity and diversity of antibiotic resistance genes in the environment, it is arguably unsurprising that resistance to every single licensed antibiotic has arisen within the first few years of clinical use (Palumbi 2001). In the following section, we review the principle mechanisms by which resistance evolves in clinical populations of bacteria.

### Resistance evolution *de novo*

Shortly after the discovery of penicillin, Abraham et al. (1941) demonstrated that cultures of *Staphylococcus aureus* could be made resistant by continuous subculture in the presence of the antibiotic *in vitro*. Because of the large mutation supply rate in some bacterial populations, the evolution of microbial resistance via random mutations is often seen experimentally or during clinical treatment (MacLean et al. 2010a). The first signs of *de novo* antibiotic resistance of clinical relevance were observed in *Mycobacterium tuberculosis* soon after the introduction of streptomycin in infected patients (Crofton and Mitchison 1948; Youmans and Williston 1948). It was later found that resistance most often arose from mutations in the ribosomal proteins, the cellular target of the antibiotics (Gillespie 2002).

*De novo* evolution of resistance is often caused by either a modification of the antibiotic's cellular target (Spratt 1994) or by increased expression of certain genes, such as those coding for efflux pumps. Expression may change due to mutations in expression pathways (Ahmetagic and Pemberton 2011; Suzuki et al. 2014) or gene amplification (Sandegren and Andersson 2009; Sun et al. 2009a). Modification of cellular targets is especially common when the genes encoding the targets are large, increasing the likelihood of spontaneous beneficial mutations affecting the target (MacLean et al. 2010a). Resistance mutations due to increased expression of cellular machinery tend to confer resistance to multiple antibiotics, especially for efflux pumps, and can be difficult to identify (Suzuki et al. 2014). High-throughput sequencing now makes it possible to track the evolution of resistance during single infections and to identify possible interactions between resistance mutations and other traits (Lieberman et al. 2011).

Because the evolution of resistance depends greatly on the mutation supply rate (i.e., the combined effect of population size and mutation rate), mechanisms that increase the mutation rate are likely to increase the rate of resistance evolution as well. Many mutations, especially those affecting the fidelity of DNA replication and repair, can cause hypermutability (Denamur and Matic 2006). Even though deleterious mutations usually outnumber beneficial mutations, an increased rate of mutation may promote adaptation in stressful environments, where fluctuating selective pressures favor phenotypic change (Taddei et al. 1997; Tanaka et al. 2003). For this reason, mutator populations of bacteria are often associated with antibiotic resistance (Björkman et al. 2000; Chopra et al. 2003; Oliver et al. 2004; Macía et al. 2005; Daurel et al. 2007; Henrichfreise et al. 2007). Interestingly, some antibiotics have mutagenic properties and therefore increase the mutation rate of bacteria (Iyer and Szybalski 1958, 1959; Kohanski et al. 2010; Gutierrez et al. 2013). The mutagenic effect of different antibiotics is mainly associated with the induction of the SOS response, which is caused by oxidative stress and DNA damage (Radman 1975; Friedberg et al. 2002).

### Rapid adaptation to new environments

In many infections, the emergence of antibiotic resistance involves the action of specialized traits that were already present in the environmental populations of bacteria. Soon after the discovery of penicillin, Abraham and Chain (1940) described an instance of such rapid adaptation when they observed an enzyme capable of inhibiting the activity of penicillin. Following the introduction of penicillin in London's hospitals, resistant strains of *S. aureus* repeatedly appeared and caused treatment failure (Barber 1947). The penicillin-resistant strains, most often harboring penicillinase activity, quickly outnumbered penicillin-sensitive strains in most hospitals, and penicillin ceased being the drug of choice to treat the bacterium (Barber and Rozwadowska-Dowzenko 1948; Nichols and Needham 1949; Rountree and Thomson 1949).

The story repeated itself countless times with resistance emerging in hospitals following the introduction of each new antibiotic. For example, aminoglycoside kinases, a large group of enzymes modifying the structure of aminoglycoside antibiotics, were discovered after the introduction of streptomycin, the first antibiotic discovered through a directed search for an antimicrobial (Davies 1994). Efflux pumps, one of the last major groups of resistance genes to be discovered, were first associated with tetracycline resistance in the 1970s (Ball et al. 1980; McMurry et al. 1980). Soon after, other efflux mechanisms in a variety of organisms were found that conferred resistance to different antibiotics. Efflux pumps remain one of the most common forms of antimicrobial resistance (Poole 2005). Over the past sixty years of research, multiple variants of each resistance mechanism group have been described.

Multidrug-resistant bacteria first emerged in European hospitals. As early as 1952, it was reported that most infectious staphylococcal strains were resistant to penicillin and tetracycline (Rountree and Thomson 1949; Clarke et al. 1952; Lowbury et al. 1952; Kirby and Ahern 1953). Today, it is estimated that between 20% and 80% of healthcare-associated infections worldwide result from multiple drug-resistant bacterial infections (Levy and Marshall 2004).

### Horizontal gene transfer

The rapid evolution of drug resistance, and multidrug resistance in particular, highlights the important role of horizontal gene transfer in microbial evolution (Ochman et al. 2000). Unlike meiotic sex in eukaryotes, genetic exchange of DNA fragments in bacteria is unidirectional and independent of reproduction (Redfield 2001; Vos 2009). Genetic material, including drug-resistant genes, can spread from one bacterium to another through plasmids, bacteriophages, and transposons [for a comprehensive review, see Thomas and Nielsen (2005)]. Although many of these mobile genetic elements can in theory evolve somewhat independently from other genes (e.g., as selfish genetic elements), many carry traits that benefit their hosts (Rankin et al. 2011). In fact, many of the most common resistance genes found in hospitals today are encoded on small plasmids that can be exchanged among different bacterial strains and species (Bennett 2008). In bacteria, chromosomal genes can also be transferred via homologous recombination or transformation, the uptake of naked DNA in the environment (Vos 2009). Transformation is believed to have enabled the evolution of penicillin-resistant *Streptococcus pneumoniae*, an important pathogenic bacterium, through the acquisition of genes from *Streptococcus viridans*, a naturally occurring penicillin-resistant bacterium (Spratt 1994). Transformation can also promote multidrug resistance, especially in the presence of standing genetic diversity (Perron et al. 2012). The capacity of microbes to acquire resistance genes from their surroundings highlights the potentially important role of environmental reservoirs in the spread of resistance across ecological niches (Perron et al. 2008a; Forsberg et al. 2012; Finley et al. 2013).

## Drug resistance as a model system for the study of evolutionary biology and ecology

Most research on antimicrobial resistance in the 20th century occurred in clinical laboratories. However, research on antimicrobial resistance led to important advances in genetics, evolutionary biology, and ecology. In the following section, we highlight classic experiments that have not only advanced clinical treatment but also unveiled the complexity of microbial evolutionary genetics and community ecology.

### Mutations and their fitness effects

How do organisms evolve over time? The answer to one of the most fundamental topics in evolutionary biology and genetics came from experiments with bacteria and the small viruses that infect them, bacteriophages. By showing that bacteria could develop resistance to bacteriophages at a predictable rate linked to bacterial replication, Luria and Delbruck concluded that spontaneous mutations caused phenotypic evolution (Luria and Delbruck 1943). Later experiments confirmed that the same mutational process explained resistance to penicillin in different species of bacteria (Demerec 1948) and that mutations in fact arise from random processes rather than selective induction (Miller and Bohnhoff 1950; Lederberg and Lederberg 1952). Early study of antibiotic resistance mutations also demonstrated that the number of mutations appearing at every generation (i.e., the mutation rate) and the differences in individual mutations’ effects on phenotype (i.e., the distribution of fitness effects) could influence the rate of resistance evolution and therefore the time for a population to evolve specific traits (Tatum and Perkins 1950; Demerec and Hanson 1951). These findings provided the first experimental support for the ‘random’ character of evolution first advanced by Darwin (1859) and espoused as a fundamental principle of evolutionary biology in the Modern Synthesis (Merlin 2010).

More recently, observations of antibiotic-resistant phenotypes and genotypes have illuminated how the distribution of fitness effects shapes evolution. Much theoretical and experimental research has attempted to describe the distribution of fitness effects of randomly generated mutations (Orr 2002, 2003, 2005; Rozen et al. 2002; Barrett et al. 2006). Experiments on antibiotic resistance evolution demonstrated that although the distribution of mutations’ fitness effects can be predictable (Kassen and Bataillon 2006), it is also sensitive to the specific dose and drug target (MacLean and Buckling 2009), suggesting there could be ways to design treatment strategies that minimize resistance evolution. The impact of horizontal gene transfer on the distribution of fixed beneficial mutations, however, remains to be investigated (MacLean et al. 2010a).

### Pleiotropy

Early work on resistance revealed that most antibiotic-resistant bacteria grow more slowly than susceptible bacteria, a phenomenon then called ‘loss of fastness’ (Todd et al. 1945; Blair et al. 1946; Eriksen 1946; Miller and Bohnhoff 1947; Klimek et al. 1948). This fitness cost, manifested as a reduced competitive ability in the absence of the antibiotic (Andersson 2006), often arises from the effects of resistance mutations on the function, stability, and folding of the protein targeted by the antibiotics (DePristo et al. 2005; MacLean et al. 2010a). It is now established that the frequency of antibiotic resistance in a bacterial population depends on both the probability of resistance arising and its pleiotropic fitness cost (Andersson and Levin 1999). In fact, this kind of trade-off between traits was a defining feature of Darwin's view of evolution: ‘The whole organism is so tied together that when slight variation in one part occurs, and are accumulated through natural selection, other parts become modified’ (1859). Of course, pleiotropy can also occur when two or more drugs have a similar target. In this situation, the effect of one resistance mutation often affects resistance to the second antibiotic. Schenk et al. (2015) describe in this issue a directed evolution experiment to test this prediction. The authors demonstrate that pleiotropic effects can shape the evolution of drug resistance in the presence of two antibiotics.

Antagonistic pleiotropy, where a single mutation has both beneficial and detrimental effects, can cause diverse outcomes in the evolution of antibiotic resistance in experimental and clinical populations of bacteria. Two meta-analyses in this issue, Melnyk et al. (2015) and Vogwill and MacLean (2015), synthesize recent research on the topic. Examining the impact of resistance mutations across a range of bacterial species and antibiotics, Melnyk et al. (2015) find that although mutations with little to no cost exist, most resistance mutations are in fact costly. This suggests that no-cost resistance mutations lead to little resistance in absence of the antibiotic and that other factors, such as compensatory mutations, play an important role in ameliorating fitness costs. The authors also find that mutations that confer more resistance tend to be more costly. However, no-cost mutations are found in clinical settings (Borrell et al. 2013), which the authors attribute to epistatic and environmental interactions. This makes it difficult to predict which resistance mutations will arise and persist in infections. Interestingly, Hall et al. (2015), also in this issue, argue that resistance costs are best understood through their effects on individual traits. Distinguishing the effects of resistance on bacterial growth and competitive ability, for instance, should lead to more accurate predictions on the spread of resistance in different environmental conditions and treatment regimens. Finally, Vogwill and MacLean (2015) find evidence of both epistasis and antagonistic pleiotropy in their analysis of multiple genotypic datasets. Resistance acquired from chromosomal mutations tends to be costlier than resistance conferred by plasmids, while plasmids conferring resistance to a wider range of antibiotics tend to be more costly than those with a narrow range. These results again illustrate the importance and subtlety of genetic interactions in the evolution of resistance.

### Epistasis

Studies on the costs of resistance have also contributed to our understanding of evolutionary compensation, that is, the attenuation of fitness costs via mutations at secondary sites that reduce or nullify the pleiotropic effects (Maisnier-Patin et al. 2002; Andersson 2006). Although compensatory adaptation can occur by reversion to the antibiotic-susceptible wild type via back mutation at the same locus (Klein and Kimmelman 1946; Miller and Bohnhoff 1950; Whitlock et al. 2003), it is estimated that such events are rare and that compensatory mutations more often alleviate the costs of resistance (Whitlock et al. 2003; Andersson 2006). As expected, compensatory mutations have been documented extensively in natural and experimental populations of bacteria (Schrag et al. 1997; Björkman et al. 2000; Reynolds 2000; Maisnier-Patin et al. 2002; Maisnier-Patin and Andersson 2004; Gagneux et al. 2006; Perron et al. 2010; Comas et al. 2012).

The presence of conditionally beneficial mutations raises interesting questions about the role of epistasis in evolution (Hall and MacLean 2011; Angst and Hall 2013). Epistasis occurs when the impact of a mutation on fitness depends on the genotype in which it occurs (Lehner 2011). It is frequently conceptualized in terms of rugged fitness landscapes (de Visser and Krug 2014). For example, detailed study of a β-lactamase gene showed that the selective accessibility of mutational trajectories for acquiring resistance depends on the genetic background and the order in which mutations appear (Weinreich et al. 2005, 2006; Schenk et al. 2013). Similarly, Hall and colleagues found that compensatory mutations allowed access to higher fitness peaks in the absence of antibiotics, enabling the bacteria to cross what was otherwise a fitness valley (Hall et al. 2010). In partial contrast, recent studies have shown that in an adaptive walk to a single fitness peak (corresponding to the resistant phenotype), the first mutation usually carries the largest phenotypic effect and the effects of subsequent mutations diminish as they accumulate (Maclean et al. 2010b; Chou et al. 2011; Khan et al. 2011). This phenomenon of ‘diminishing returns’ (de Visser et al. 1999) suggests that the rate of phenotypic adaptation may be predictable to some extent (Kryazhimskiy et al. 2014), as suggested by Fisher's fundamental theorem of natural selection (Fisher 1930).

The evolution of resistance to most first-line antibiotics spurred the use of additional antibiotics and combination therapies (Levy and Marshall 2004). Combination therapies have revealed how resistance mechanisms can facilitate resistance to other antibiotics or, in some cases, reduce the likelihood of resistance to a second antibiotic. Early evidence of epistasis between resistance mechanisms was mostly inferential and anecdotal (Perron et al. 2008a), but more extensive screening of resistance mutations in experimental microcosms has revealed complex interactions between different mutations (Trindade et al. 2009; Ward et al. 2009). In natural population of bacteria, epistasis between antibiotic resistance mutations can drive the evolution of multidrug-resistant tuberculosis that suffers little or no fitness cost (Borrell et al. 2013). Horizontal gene transfer also increases opportunities for advantageous gene combinations to arise and spread in the population (Silva et al. 2011).

### Evolutionary ecology

The shortage of new antibiotics motivated the search for novel strategies to manage resistance evolution. For example, combination therapy showed that some antibiotics have nonlinear inhibitory activities when used together (King et al. 1981; Yeh et al. 2009). Extensive phenotypic screens revealed that many classes of antibiotics could inhibit the effects of other antibiotics given their modes of activity (Yeh et al. 2006), often caused by regulatory conflicts in the expression of the antibiotics’ cellular targets (Bollenbach and Kishony 2011) or by reductions in bacterial growth rates (Ocampo et al. 2014). Subsequent studies have shown that such interactions could modulate resistance evolution (Yeh et al. 2009): Multidrug resistance is unlikely to evolve against sets of antibiotics that inhibit each other's activity, as resistance evolution to one antibiotic would expose the bacterium to the full efficiency of the second antibiotic (Michel et al. 2008). However, additional clinical trials are required to see whether this finding can be put into practice.

Multidrug resistance can also be compared to the evolution of generalists. A central question is how antibiotic regimes favor or inhibit antibiotic resistance (Bonhoeffer et al. 1997; Lipsitch et al. 2000). Although population genetics theory suggests that cycling different antibiotics is likely to select for the evolution of a multidrug-resistant ‘generalist’ bacteria (Bergstrom et al. 2004; Levin and Bonten 2004), optimization theory suggests that there should always exist a cycling regime that minimizes resistance evolution (Beardmore and Peña-Miller 2010). However, the parameters to test such concepts are hard to identify, and observations give mixed results (Kollef 2001; Warren et al. 2004; Curtis 2009). The feasibility of antibiotic cycling as an alternative to combination therapy is further complicated by the fact that evolutionary trajectories may be sensitive to the order in which antibiotics are used (Perron et al. 2007; Kim et al. 2014) and epistasis between resistance (and other) mutations that accumulate over treatment (Imamovic and Sommer 2013).

The strengths of selective pressures imposed by antibiotic and antibiotic concentrations also affect resistance evolution. In a classic work, Drlica suggested that only selective pressures of intermediate strength could lead to resistance evolution (Drlica 2003): Antibiotic concentrations needed to be high enough to impose selective pressures on rare resistant mutants, but not too high as to prevent the growth of any mutants. However, a series of experiments demonstrated that a slow increase in antibiotic concentration can lead to resistance evolution outside the selection window (Perron et al. 2006, 2008b; Lindsey et al. 2013), favoring evolutionary rescue during treatment (Gonzalez et al. 2013). Lindsey and colleagues demonstrated that higher rates of environmental change made certain genotypes evolutionarily inaccessible (Lindsey et al. 2013), possibly affecting further evolution of the trait. Finally, Zhang and colleagues showed that heterogeneous environments favored resistance evolution when dispersal exposed bacteria to different concentrations of antibiotics (Zhang et al. 2011).

## Discussion

### Evolutionary applications

Given the seemingly unavoidable nature of antibiotic resistance, evolutionary biologists have a large role to play in microbial resistance management. A better understanding of resistance evolution could not only help extend the use of antibiotics but could also help identify future antibiotics and strategies that may be more successful (Bush et al. 2011). For example, models based on community ecology could uncover how resistance is influenced by competition, migration, and environmental conditions. This direction is especially important given the accumulating evidences for the contribution of resistance genes already present in nature to the evolution of novel traits (Martinez 2012; Wellington et al. 2013).

Evolutionary biologists can further understanding of fitness costs of resistance in many ways. This issue contains several articles on this topic, from the distributions of fitness costs in experimental populations (Melnyk et al. 2015) and the costs’ associations with their genetic basis (Vogwill and MacLean 2015), to the relationship between costs of resistance and strain backgrounds in different environments (Hall et al. 2015) and the pleiotropic effect of resistance mutation in the presence of multiple antibiotics (Schenk et al. 2015). This information is crucial to predict the distribution and persistence of antibiotic-resistant bacteria.

More generally, understanding the evolutionary and community ecology of drug resistance is essential for successful long-term management of antibiotic-resistant infections. The introduction of penicillin helped cure infections caused by *Streptococcus* but potentially enabled other bacteria, such as *S. aureus*, to fill the vacant niche (Levy and Marshall 2004). More recently, multiple-antibiotic-resistant bacteria such as *Clostridium difficile* have spread in hospitals around the world, most often infecting patients treated with antibiotics. Other opportunistic pathogens that can evolve multidrug resistance, such as *Pseudomonas aeruginosa* and *Acinetobacter baumannii*, have increased in frequency in hospitals (Chen et al. 2008; Giske et al. 2008). Future treatment strategies should consider how host microbial communities affect patients’ resistance to infections (Lemon et al. 2012).

The study of social interactions in microbes also shows great promises. Excreted proteins can act as public goods (Griffin et al. 2004), meaning that extracellular resistance mechanisms can contribute to the protection of whole communities (Dugatkin et al. 2005). Such effects can maintain resistance plasmids in experimental populations of bacteria (Yurtsev et al. 2013) and confer protection to unrelated bacteria species (Perlin et al. 2009). Applying principles of social evolution could lead to new treatments (Boyle et al. 2013). For example, antivirulence drugs are an especially promising method to control bacterial infections. Rather than trying to kill bacteria, these drugs modulate social interactions between microbes, selecting for less virulent variants (Brown et al. 2009; Ross-Gillespie et al. 2014). For more discussion of the role of evolutionary biology in managing antibiotic resistance in human medicine and agriculture, refer to Baquero et al. (2015) and Chang et al. (2015), respectively.

## Coda

Here, we have focused on antimicrobial resistance in bacteria, one of the most dramatic case of contemporary evolution (Maynard Smith et al. 2000). However, we must emphasize that resistance is a widespread phenomenon impacting many other important activities in medicine and agriculture. Notable examples include resistance in viruses such HIV (Fauci 2003), pests such as mosquitoes (Georghiou 1983) and weeds (Holt and Lebaron 1990), and cancer cells (Cunningham et al. 2004). This issue includes one example of resistance not affecting bacteria: the evolution of malaria to artemisinin (Pollitt et al. 2015). Pollitt and colleagues demonstrate that, like in many bacterial populations, standing genetic diversity leads to rapid drug resistance in natural population of murine *Plasmodium*. This example highlights the strong parallel between drug resistance in bacteria and other parasites. Articles in this issue showcase the insights that evolutionary biology can offer public health while providing ample examples of the utility of resistance evolution as a model system in ecology and evolution.
